# The development of new remote technologies in disaster medicine education: A scoping review

**DOI:** 10.3389/fpubh.2023.1029558

**Published:** 2023-03-24

**Authors:** Chia-Lung Kao, Li-Chien Chien, Mei-Chin Wang, Jing-Shia Tang, Po-Chang Huang, Chia-Chang Chuang, Chung-Liang Shih

**Affiliations:** ^1^Department of Emergency Medicine, National Cheng Kung University Hospital, College of Medicine, National Cheng Kung University, Tainan, Taiwan; ^2^Regional Emergency Medical Operations Center-Tainan Branch, Ministry of Health and Welfare, Taipei City, Taiwan; ^3^Department of Nursing, Chung Hwa University of Medical Technology, Tainan, Taiwan; ^4^Department of Medical Affairs, Ministry of Health and Welfare, Taipei City, Taiwan; ^5^Department of Emergency Medicine, College of Medicine, National Taiwan University, Taipei City, Taiwan

**Keywords:** remote learning, remote technology, AI pedagogical approach, disaster medicine (DM), mobile edge computing (MEC), head-mounted display (HMD)

## Abstract

**Background:**

Remote teaching and online learning have significantly changed the responsiveness and accessibility after the COVID-19 pandemic. Disaster medicine (DM) has recently gained prominence as a critical issue due to the high frequency of worldwide disasters, especially in 2021. The new artificial intelligence (AI)-enhanced technologies and concepts have recently progressed in DM education.

**Objectives:**

The aim of this article is to familiarize the reader with the remote technologies that have been developed and used in DM education over the past 20 years.

**Literature scoping reviews:**

Mobile edge computing (MEC), unmanned aerial vehicles (UAVs)/drones, deep learning (DL), and visual reality stimulation, e.g., head-mounted display (HMD), are selected as promising and inspiring designs in DM education.

**Methods:**

We performed a comprehensive review of the literature on the remote technologies applied in DM pedagogy for medical, nursing, and social work, as well as other health discipline students, e.g., paramedics. Databases including PubMed (MEDLINE), ISI Web of Science (WOS), EBSCO (EBSCO Essentials), Embase (EMB), and Scopus were used. The sourced results were recorded in a Preferred Reporting Items for Systematic Reviews and Meta-Analyses (PRISMA) flowchart and followed in accordance with the PRISMA extension Scoping Review checklist. We included peer-reviewed articles, Epubs (electronic publications such as databases), and proceedings written in English. VOSviewer for related keywords extracted from review articles presented as a tabular summary to demonstrate their occurrence and connections among these DM education articles from 2000 to 2022.

**Results:**

A total of 1,080 research articles on remote technologies in DM were initially reviewed. After exclusion, 64 articles were included in our review. *Emergency remote teaching/learning education, remote learning, online learning/teaching, and blended learning* are the most frequently used keywords. As new remote technologies used in emergencies become more advanced, DM pedagogy is facing more complex problems.

**Discussions:**

Artificial intelligence-enhanced remote technologies promote learning incentives for medical undergraduate students or graduate professionals, but the efficacy of learning quality remains uncertain. More blended AI-modulating pedagogies in DM education could be increasingly important in the future. More sophisticated evaluation and assessment are needed to implement carefully considered designs for effective DM education.

## Introduction

The emerging evolution of remote technology in disasters has been developed in a multidisciplinary manner and has progressed tremendously in completely different settings, e.g., the COVID-19 pandemic and armed conflicts (1–4). The pandemic has changed the paradigm of traditional face-to-face classroom education and contributed to technology-driven pedagogies such as digital transformations and distance learning through hybrid telecommunications (5–7). The changes are not only affecting postsecondary student pedagogies in developed countries but also in developing countries such as the Southern African Customs Union (SACU) (8–10).

Over the last 20 years, the occurrence, impact magnitude, and economic loss from disasters have increased significantly and show an upward trend worldwide (11). According to the 2022 Disasters Year in Review (Emergency Event Database, EM-DAT), published by the Center for Research on the Epidemiology of Disasters (CRED), the category *natural hazards*, e.g., *flood, storm, and earthquake*, had dominated the *major events* of 2021, and the annual occurrence records are higher than those for the period covering 2001–2020. A total of 432 catastrophic events were recorded in 2021, which is significantly higher than the average of 357 annual catastrophic events for the 2001–2020 average. The catastrophic events of 2021 increased significantly compared to the previous two decades and resulted in extensive economic losses (Supplementary Data). In 2021, the *disastrous events* accounted for 10,492 deaths, 101.8 million people affected, and 252.1 billion USD in economic losses (12). Asia, especially China and India, remain the most severely impacted countries and has nearly half of the total number of mortalities and 66% of the total wounded. Northern America, especially the US, was severely affected by floods, storms, and the COVID-19 pandemic in 2021, which led to extensive economic losses of up to 112.5 billion USD. Furthermore, Europe was affected by unexpected cold and heat waves in 2021. The severe cold in France in April and wildfires in the Mediterranean regions (Algeria, Bulgaria, Cyprus, Greece, Italy, Macedonia, Tunisia, and Turkey) during the summer caused substantial agricultural damage with devastating consequences (13). The need for disaster medicine education to adequately prepare undergraduate students or graduate professionals for these kinds of disasters will require novel technologies, as well as traditional ones, to meet this challenge.

Due to the advent of artificial intelligence (AI) that relies on big data and the proliferation of the Internet of Things (IoT) for universal applications, e.g., smartphones, smart industries, and smart healthcare, wirelessly powered communication (WPC) devices and distant healthcare delivery systems are booming (14, 15). Mobile edge computing (MEC), a recently developed concept since 2014, is an emergent architecture where cloud computing services are extended to the edge of networks leveraging mobile base station (16). *Edge computing* refers to a broad set of techniques designed to move computing and storage out of the remote cloud (public or private) and closer to the source of data (17), i.e., the computing resources could be sent to the network's edge nearby the end-mobile devices (18). The MEC could not only efficiently perform emergency computational tasks but also provide advanced, realistic DM training. For example, unmanned aerial vehicles (UAVs) are deployed as flying IoT base stations (UAV-enabled sensors network) in order to enhance communication coverage and facilitate real-time data in the affected areas after disasters (19, 20). UAVs equipped with sensors have versatile uses from detecting toxic pollution to providing a 3-D realistic simulation to enabling an education scenario for first responders in disasters (21). Moreover, these UAVs equipped with miniaturized sensors could also support special missions such as sampling and identification of chemical, biological, radiological, and nuclear (CBRN) events for either civilian organizations or military forces (e.g., NATO Defense Alliance) (22). The significant technological development in mission-oriented UAVs could not only perform detection, identification, and monitoring of CBRN events in affected areas but could also be used as a useful tool for offering postgraduate courses in CBRN protection (22). Digital devices have transformed from “networked computers for collaborative learning” in the 1997–2006 period to “online digital learning” in the 2007–2016 period (23, 24). By using a high-fidelity immersive head-mounted device (HMD) designed by virtual/augmented reality (VR/AR) or newly developed mixed reality (MR)/HoloLens technology, along with disaster risk big data, we can easily build more comprehensive scenarios and learning models for trainees while facing disasters (25). The AI-enhanced training platform is a feasible alternative for undergraduate medical students, professionals, or ordinary people to be familiar with pre-emergency preparedness, disaster assessment, or emergency response/evacuation in simulated settings rather than dangerous environments, e.g., fire, pandemic, earthquake, or toxic situations (26, 27). The HMD-based teaching tools were found to be more effective and viable than other digital devices in medical education (28). Extended reality (XR)-based HMD utilizes the concepts of AR, VR, and MR and could provide more beneficial effects on medical skills and knowledge in medical training (29).

Even though the immersive HMD could make it easier to come up with a new and interesting way for medical students to learn, the DM curriculum should be implemented using an evidence-based, multi-modal approach because training programs do not give students enough opportunities to do cooperative activities, group work, and social interaction at the same time (30). We will discuss HMD-based immersive VR (IVR) in DM education regarding new developments, training efficacies, and how participants value their experience with these technologies.

An extensive literature review was conducted in order to provide a cutting-edge evaluation of the development of medical school students' or health professionals' learning by using remote technologies in facing disasters or emergencies. This review checked different remote learning systems under development, the teaching effects of different applications, and the possible changes in the pedagogy of DM that could materialize in the future. This review focuses on the newly developed technologies applied in DM education and training programs for medical and other undergraduate students. The four specific queries are as follows:

1. What kinds of newly remote technologies have been developed in DM and DM education?
2. What are the related articles or keywords described in the field of DM education in recent years?
3. Could remote technologies adopted in learning pedagogies and training programs for medical staff improve their capabilities?
4. What future objectives will new DM learning attain or strive for?

## Research methodology

### Searching strategies and inclusion and exclusion criteria

Five major electronic databases, including PubMed database (MEDLINE), ISI Web of Science (WOS), EBSCO Essentials (EBSCO), EMBASE (EMB), and Scopus, from 1 January 2000 to 15 May 2022 were compiled in our review. All articles were from the Science Citation Index (SCI) and Social Science Citation Index (SSCI) databases. After the literature was reviewed, we obtained the related keywords with the high number of occurrences as queries with the following terms: *remote technology* (6, 31, 32); *remote learning* (6, 31, 33); *online learning or teaching* (7, 10); *emergency or disaster response* (34, 35); *disaster medicine education* (30, 36); *blended learning* (37–39); *mobile edge computing* (20, 40); *virtual or augmented reality* (23, 25, 41, 42); *drone* (14, 20, 43); *machine learning* (44, 45); *deep learning* (46, 47); and *federated learning* (48, 49). Similar articles included in references were also screened. The inclusion criteria were reports or peer-reviewed studies of education and training programs, which were focused on any kind of disaster or emergency such as medical, nursing, pharmacy, social work, or paramedical science, and also for other hospital staffs such as administrators; and peer-reviewed studies, accepted articles for publication, e.g., electronic publications (Epubs), and proceedings which were written in English. Two of our coauthors screened the abstracts for potential articles, and the full texts were checked in detail to decide whether they correlated with our eligibility criteria or not. The exclusion criteria were conference abstracts, unpublished manuscripts, and whitepapers available online; non-medical issues such as industrial or geoengineering planning or projects; articles not published in English; and articles on disaster medicine but do not mention specific education or training programs.

### Data collection and bibliometric analysis

Information visualization (InfoVis) is a visual representation technique utilizing data from abstracts to allow researchers to quickly analyze and understand the huge amount of multidimensional data produced in emergencies (50). Bibliometrics is an InfoVis analysis that quantitatively and qualitatively evaluates citations to scientific publications by constructing and mapping citation graphs to a network representation (51–53). The VOSviewer is the most common tool specifically designed for constructing and visualizing research trends and the maps of co-occurrence keywords in health-related literature on natural disasters or epidemic outbreaks (54–56). The software generates network and cluster visualizations of the data, which can be useful in identifying patterns and relationships among the keywords, including the titles and abstracts of reviewed articles. Cluster Density Visualization is a vivid demonstration of the dataset, and the software will automatically be grouped in clusters according to those related keywords. High densities of connections among the keywords within a cluster will indicate that those keywords are highly related, while low densities of connections show that the keywords are less related. This representation provides a way to identify and explore the relationships between different groups of keywords. We used VOSviewer (v.1.6.18, 2022) to analyze the keywords from the semantic contents we retrieved from PubMed (reference). The keywords included “remote technology AND disaster education,” “remote learning AND disaster medicine education,” “remote learning experience AND disaster medicine education,” “remote system AND disaster medicine education,” “mobile edge computing AND disaster medicine education,” “virtual /augmented reality AND disaster medicine education,” “drone AND disaster medicine education,” and “machine learning AND disaster medicine education.” VOSviewer maps the keywords according to the frequencies and the co-occurrences. According to the circle size which is proportional to the frequencies of the keywords, we chose the remote technology-relevant keywords.

## Results

### Searching process

**Figure 1:**
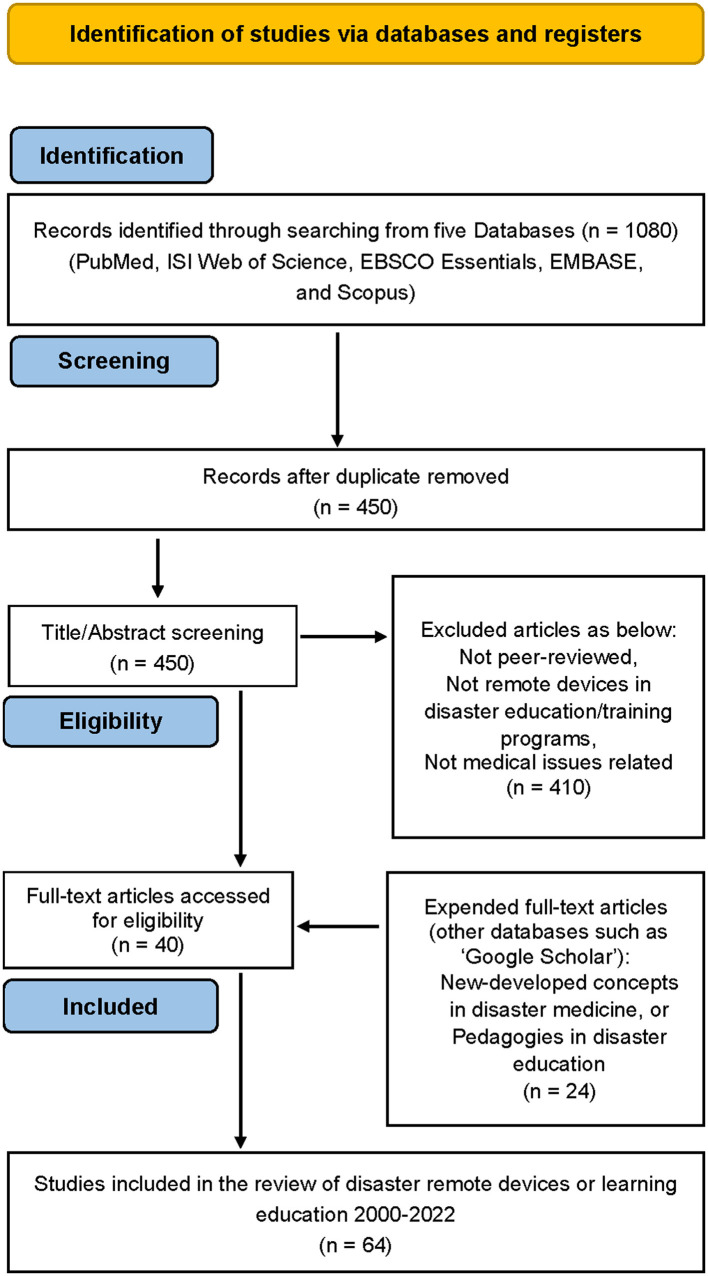
Flow of the literature search process (PRISMA chart)).

The Preferred Reporting Items for Systematic Reviews Analysis and Meta-Analyses (PRISMA) charting and PRISMA extension for Scoping Reviews (PRISMA-ScR) statement were adopted in the flow of the literature search process (57, 58). In the first, 1080 relevant articles were screened from the databases: 402 from MEDLINE, 250 from WOS, 150 from EBSCO, 134 from EMB, and 144 from Scopus (Figure 1).

Duplicated publications from different databases were checked by two of our authors manually. After duplicates were removed, 450 articles were assessed for eligibility. A further 410 articles were excluded due to failure to correlate with our stated criteria. We added the extended articles, which have been highly cited by other websites (e.g., Google) but were not shown in our previous search of databases (i.e., 24 articles extended). All of the 64 articles in the online electronic databases that were published in peer-reviewed journals are considered to be reliable.

### The changes in DM education (blended learning)

The “hybrid/blended learning” (technology-enhanced learning, problem-based learning, table-top exercise, and computerized simulations) (59, 60) core curriculum focusing on different kinds of disaster scenarios, either in-hospital (e.g., contaminated victims) or out-hospital events (e.g., landslides, floods, and earthquakes), has been implemented in a variety of DM applications and delivered to undergraduate medical or paramedic students in order to improve their disaster preparedness knowledge and the capabilities of prehospital disaster management (36, 37, 61).

### The advent of remote technologies in DM education or training program

Many related state-of-the-art technologies have been integrated into disaster rescue, risk management, epidemic tackling, and remote learning and education (19, 31, 33, 43, 62–64). We reviewed three major categories of distance learning design in DM education and we discuss them in detail below.

#### Efficient MEC technologies for DM training and education

The emergence of computation-intensive applications such as MEC devices could provide wide spatial coverage in affected areas during disasters and could perform emergency computational tasks in civil and military areas, which significantly improve post-disaster management (18, 19, 65). The MEC technologies such as webcam/photo-realistic 3-D model imagery capture (66, 67), intelligent wearable medical equipment (point-of-care sonography) (68), robotics (69), and social media (70–72) have all been shown to be beneficial in a variety of emergencies.

For instance, flying UAVs/drones integrated with a MEC server (UAV-enabled MEC), in conjunction with the Global Navigation Satellite System (GNSS), real-time computer vision, and advanced photogrammetric techniques (including deep learning algorithms), could enable first responders to obtain accurate 3-D imagery of affected areas *via* cloud computing (CC) and aid in the rapid identification of victims (73, 74). Not only could drones be used in disaster mapping for search and rescue operations but also could provide an excellent resource for training paramedic students in mass casualty incidents (MCIs) (75). In addition, 80% of nursing students in the Emergency Nursing Master program at the Catholic University of Murcia (UCAM) reported a significant improvement in their self-perception and performance in the MCI simulation with the assistance of drones (76).

#### Machine learning applied in DM education

Machine learning (ML) algorithms, such as the evidence-based medicine (EBM) movement, have become a powerful prediction tool ranging from clinical diagnosis to enhancing desired healthy behaviors (77). The medical school curriculum as well as the postgraduate medical education within academic hospitals around the world are facing the challenges of this AI-driven data science revolution (78). Among them, deep learning (DL) was developed from traditional ML techniques and provides more accurate classifications and predictions in disasters, e.g., as victims' detection, image segmentation, and medical information processing, due to powerful computing capacity and the high availability of large datasets (79–81). For example, DL trains a system to filter the digital images and to aid in the prediction of whether a corresponding patient is infected with COVID-19 or not (82). In addition, using convolutional neural network (CNN) technology, a radiologist in a remote area may determine whether a patient's lung is impacted by COVID-19 using chest X-ray images (83, 84). CNN is the most established algorithm among various DL models, which is an artificial neural network dominant in computer vision tasks by stacking mathematical layer operations, i.e., convolution (83). Innovative digital technologies were found to boost AI detection performance during the COVID-19 pandemic while also enhancing learning models for medical professionals in disease diagnosis (46, 47, 85). DL could also detect and identify large amounts of data from social media messages (urgent and not urgent tweets) during a disaster and confirmed a high efficacy in detecting urgent tweets during hurricanes, based on a combined CNN, trained on average word embedding, and support-vector machine (SVM), trained on full word embedding (71). DL not only provides a modern computational platform and automated medical practice but also refers to a comprehensive human learning approach and has been integrated into medical education (86). E-learning in teaching emergency disaster response (ELITE-DR) among undergraduate medical students could provide comprehensive levels of cognitive concepts and visual stimuli for disaster response medicine (87). However, surveys reveal few formal teaching programs incorporated into medical education about AI/ML, whether in Europe, the US, or even in Asian countries (88–91). For example, 66.5% of final year medical school students in Ireland reported zero hours of teaching on AI/ML during their degree, 43.4% had not heard of the term “machine learning,” and 80.6% had not read any academic journal articles on AI/ML (44).

The applications of AI, ML, and DL have been widely introduced and developed for disaster management, i.e., in the aspects of hazard prediction, vulnerability assessment, disaster early warning systems, disaster monitoring, damage assessment, and post-disaster response (92). The preparedness for disaster risk reduction (DRR) (93) and the training on AI for disaster management (94) are important teaching models and could improve first responders' capability while facing different catastrophes. Federated Learning (FL) approach, a de-centralized data collection technique, generates more robust models without sharing data in a central server and enables collaborative training of multiple MECs (e.g., UAVs) locally (48, 95). FL trains at distributed MEC devices to offload data in order to reduce the data crowdsensing, delay transmission, and also protect IoT application privacy (96). FL could prove a more effective training model in disaster scenarios due to massive irrelevant data and a high flow of scalable IoT networks while in disasters. The privacy-preserved federated transfer learning (FedTL) approach could also provide and verify a real disaster image dataset collected from distributed social computing nodes (97). The new-developed FL model will give both AI and humans (e.g., first responders) a sophisticated training course and achieve better performance for the medical image learning model (98).

#### HMD-IVR devices applied in DM education

Virtual reality simulation (VRS) technology is widely used and it seems to be a viable training alternative in different disciplines, e.g., a simulation realistically of different hospital disaster scenarios, which could provide positive effects of confidence and necessary knowledge acquisition for healthcare professionals in hospital (99). Due to the features of reproducibility and repeatability of VRS, the newly developed technology could also improve CBRN training courses for both military and civilian responders to CBRN events (VERTIgO project) (100). VR delivers virtual scenarios and combines with multi-source data, 360 degrees video, and intuitive interaction interfaces to facilitate quality interprofessional education (101, 102). The so-called “Just-in-time” training programs could offer effective personal preparation and resiliency by enhancing responders' situational awareness in critical overloading situations, e.g., in the COVID-19 pandemic (103). Furthermore, the serious game (SG), one of the popularly used game genres nowadays, combines practical aspects with original amusement but is implemented for health or medical education (non-entertain) purposes (104). SG has been applied in improving medical technical skills such as laparoscopic surgery or arthroscopic intervention for postgraduate residency training in medical settings (105, 106). Disaster risk management (DRM)-related SG/simulation also provides a good educational and engagement tool for affected communities, policy-makers, and other vulnerable population (107). The SGs/simulations, with the realistically simulating disaster reality, offer assistance especially in the realm of disaster risk awareness raising, identifying hazards, undertaking preventive actions, empathy triggering, and perspective-taking (107). Moreover, 3-D video capture HMDs provide a more interesting way to interact and contribute to positive feedback responses from children, which are also vulnerable groups in disaster and public health emergencies (108, 109). Although the VRS technology shows a higher learning atmosphere, comprehensibility, and overall recommendation of teaching courses than those with a non-immersive screen (110), some articles showed conflicting results on VR HMD benefits or even no benefits at all (111, 112). Among them, technology limitations (surgical training), usability challenges (less powerful hardware), cybersickness, and limited participants' induced questionable assessment were potential limitations (113).

### Bibliometric analysis for articles reviewed

**Figure 2:**
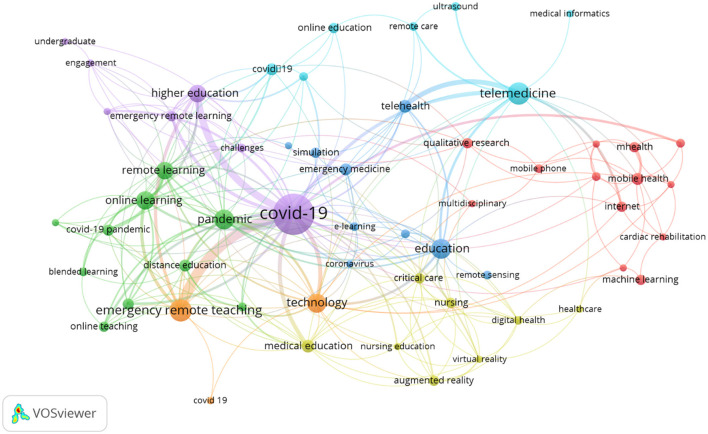
The network visualization figure of the screened keywords.

By analyzing with VOSviewer, we obtained the co-occurrence MeSH keywords mapped as shown with Network and Cluster Density Visualization of the retrieved title and abstract (Figure 2). In the network visualization figure (Figure 2), the size of the circle is proportionate to the frequency of the keywords, and larger circles at keywords mean that they appear more frequently in the dataset. The keywords are categorized into six clusters according to the highly related connection. “COVID-19” classified in cluster 6, is correlated with the keywords “online learning,” “online teaching,” “remote learning,” and “remote teaching” in cluster 1. The keyword “medical education” classified in cluster 4 also has the same correlation with the keywords “online learning,” “online teaching,” “remote learning,” and “remote teaching” in cluster 1. The keywords were classified automatically into six clusters and are presented in Table 1.

**Table 1:** Keywords classified as six clusters by VOSviewer.

**Cluster 1**	**Cluster 2**	**Cluster 3**	**Cluster 4**	**Cluster 5**	**Cluster 6**
Blended learning	Artificial intelligence	Coronavirus	Augmented reality	Challenges	COVID-19
COVID-19	Cardiac rehabilitation	e-learning	Critical care	COVID-19	Emergency remote education
COVID-19 pandemic	e-health	Education	Digital health	Emergency remote learning	Medical informatics
Distance education	Internet	Emergency medicine	Healthcare	Engagement	Online education
Emergency remote teaching	Machine learning	Public health	Medical education	Higher education	Remote care
Online learning	Mental health	Remote sensing	Nursing	Pedagogy	Telemedicine
Online teaching	M-health	Simulation	Nursing education	Undergraduate	Ultrasound
Pandemic	Mobile applications	Telehealth	Virtual reality		
Remote learning	Mobile health	Tele-simulation			
Remote teaching	Mobile phone				
Teachers	Multidisciplinary				
Teaching	Qualitative research				
Technology	Smartphone				

### Publication trends demonstrated by VOSviewer

**Figure 3:**
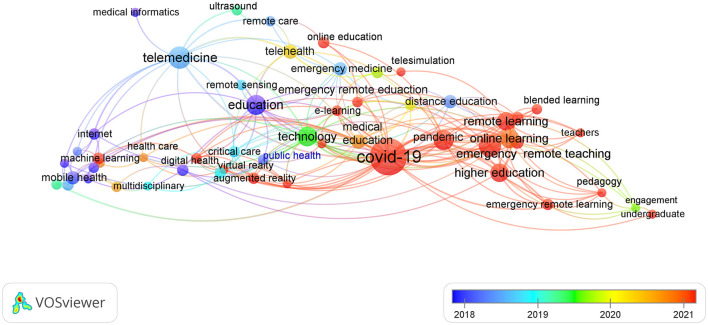
The variation of the screened keywords changed with time.

Figure 3 presents the study trend as it varied from 2010 to the present. Early in 2020, the remote equipment applied to emergency management was studied. Then, the research target moved to telemedicine and remote sensing technology with the flourishing development of communication technology. While COVID-19 outbreaks had occurred by the end of 2019, the keywords “emergency remote teaching,” “remote learning,” “online learning,” “emergency remote learning,” “blended learning,” and “emergency remote education” mirror the global education remote technology methods that were necessitated to educate the students and to keep safe social distances. Based on the correlation, it seems that people did a lot of studies related to education while they were isolated during the COVID-19 pandemic.

### Future education models proposed by AI-integrated platform

**Figure 4:**
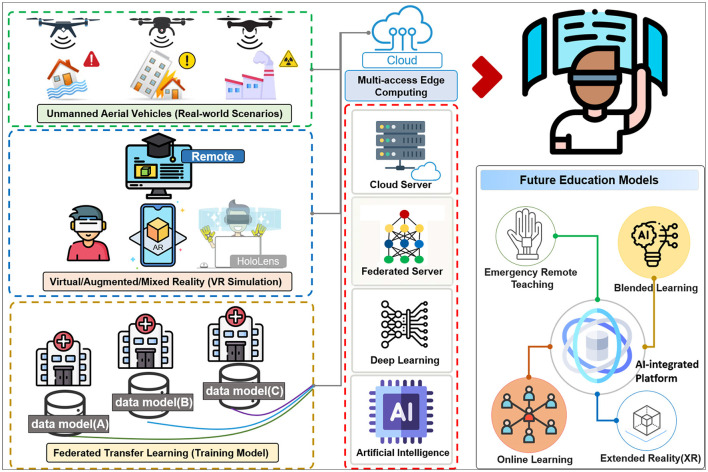
The integration of real-world scenarios with artificial intelligence technologies initiates disaster medicine remote learning (Future Education Models).

Figure 4 shows the current application technology and the future trend of DM education. Using UAVs and photography equipment to obtain real disaster scenes and recreating them using VRS technologies, such as VR, AR, and MR technologies can provide students with a more authentic learning experience. These technologies can be used to simulate real-world scenarios and provide a more immersive experience, which can help students better understand and prepare for potential disasters.

Big data analysis and advanced AI technologies, such as deep learning and federated learning, can also be used in disaster management education. For example, these technologies can be used to analyze large amounts of data from past disasters and identify patterns and trends that can inform future disaster management strategies. They can also be used to develop more accurate simulations and prediction models, which can be used to train students and prepare them for real-world scenarios.

Integrating and applying these new technologies can also enable new educational models, such as remote learning and online teaching, which can increase access to disaster management education and make it more convenient and flexible for students. Overall, the use of technology in disaster management education can significantly enhance students' learning experiences and prepare them more effectively for real-world scenarios.

## Discussion

Blended learning in DM education has been implemented significantly by undergraduate medical or paramedic students and applied widely by clinical professionals. The occurrence of the COVID-19 global epidemic has significantly precipitated the progress and implementation of integrated hybrid (including station rotation, simulation, and distance models) learning in higher education and also in DM education (30, 39). Implementing special epidemic training for medical students, such as COVID-19 pandemic simulated programs, could significantly improve their preparedness, knowledge, and skills when dealing with emerging infectious diseases (114). The use of IoT, social media, UAVs, and advanced machine learning is not only engaging in rapid response, management, and risk reduction in disasters (DRR; e.g., Sendai Framework for Disaster Risk Reduction 2015–2030) but also stressing the important governance challenge of multidisciplinary, transdisciplinary, and interdisciplinary teaching courses on DRR implemented in many universities (115). The remote e-learning DM programs appear to be a crucial concern, and medical schools in vulnerable regions such as Asian countries or low- and middle-income countries, as we previously indicated, need to continue teaching emergency medicine (10, 33). The inability of these countries to afford state-of-the-art technologies (such as VRS-VR or AR) or to have a convenient AI network prevents medical students from using AI to further medical education. In addition to multidisciplinary approaches used in DRR, more inclusive curricula, a theoretical emphasis, a field orientation, and skill development appear to be essential for higher education. However, due to the popularity of AI-enhanced remote learning modalities among medical school students, it is important for faculty to be well-versed in online learning techniques, e-Learning tools, and cutting-edge technology (such as HoloLens) before classes. The new disruptive technologies based on integrated AI, big data, blockchain, and VR/AR could enrich teaching formats and provide broader guidance for faculty resource allocation in pedagogical methods (19, 115). Our review focuses on the application of remote technology in DM education rather than discussing the educational methods or content of different trainee levels. However, strict quality assurance methods (such as Quality Matters) (116) and continuous quality improvement (CQI) should be closely observed to enable faculty to quickly adjust to changes under the guidance of well-defined implementation plans (115).

We evaluated UAV-enabled MEC with 3-D imagery and found it useful for a variety of detection techniques in remote or disaster rescue operations. However, some restraints on the UAV during operations exist, e.g., the huge operational expenditure to maintain its infrastructural nodes or battery endurance for longer aerial communication. A “UAV-enabled wireless powering IoT” (Ue-WPIoT) with smart trajectory information of UAVs and an AI-deep learning model was developed to overcome the limitations and was designed for the consideration of more lightweight UAVs (14). The improvement of Ue-WPIoT could allow trainees more time to become familiar with the new device and fulfill the IoT wireless function in the air. In addition, a new system called the Aerial Remote Triage System has changed how MCI triage is usually done so that life-saving interventions and transportation can happen more quickly (117).

With the development of telecommunication technology, the fifth generation (5G) remote network significantly increased the band and the speed of data transmission, which was very helpful for long-distance medical diagnosis. In the field of DM, telemedicine benefited from remote devices related to research that were popular before the pandemic COVID-19. Remote learning-related research became the hottest study topic for conducting medical education corresponding to the COVID-19 isolation policy. Based on the well-established communication infrastructure and rapidly progressing telecommunication technology, we anticipate that future research will focus on the application of wearable devices in fields such as medical care and monitoring, educational instruments, and emergency rescue training programs.

The Internet of Things has been formulated to establish wireless sensor networks, AI-enhanced monitoring, and smart embedded devices (ex. in UAVs) to facilitate crowd-sourcing (CS) communication, IoT-based Early Warning System (IoTEWS), efficient victim localization (RFID tag and topology preserving maps), data analytics, and knowledge aggregation (effective data mining of collected big data) in disaster management (118, 119). Moreover, the concept of the Internet of Everything (IoE) or even the Internet of Nano Things (IoNT) was built to account for everything that could be connected to the Internet, i.e., it facilitated the connections among people, processes, data, and things (“four pillars”). The IoE provides the potential to analyze millions of connected sensors' data in order to aid automated- and people-based processes. The application of IoE could provide a remote platform by the expansion of CC to connect “everything” online with the implementation of machine-to-machine (M2M), machine-to-people (M2P), and people-to-people (P2P) (120). The concept of IoNT is being extended from IoE to achieve nanoscale networks by embedding nanosensors in diverse objects such as wearable medical devices and by connecting conventional microsensors to nanonetworks (121). Using nanodevices embedded in the environment and medical equipment, the IoNT supports computing for end users to improve in-time data monitoring and early disease diagnosis. Moreover, the IoT could provide positive implications for computer science education by reshaping web programming, tremendous excitement from online teaching, and real-world sensing applications, performed by UK Open University according to IoT principles in remote lessons for more than 2000 students in 2012 (120, 122).

Our review indicates that the newly emergent information technologies, including IoT, Big Data, social media, and machine learning, could facilitate DM tasks in visualizing, analyzing, and predicting disasters and improve the resilience of communities (123–125). Using Big Data Analytics tools and social media data mining (e.g., large-scale satellite imagery data mining) could empower all sectors, from citizens to community, government to non-government organizations, effective remote communication networks, and knowledge graphs. Remote technologies could also provide an integrated conceptual approach to higher education, such as in massive open online courses (MOOCs) or Flipped Classroom (126, 127). However, the AI innovation in DM education programs did not turn out as planned (44, 89, 128). To address the various ways in which medical students and instructors learn and receive feedback from customized lessons, more AI-guided learning pathways need to be developed. Furthermore, assessing the effectiveness of AI teaching modality and their progress in AI technologies, such as IVR, also require AI-mediated pathways (129).

As previously stated, immersive HMDs have many beneficial potentials in disaster scenarios, and there has been a lot of research interest in DM education program applications (23). VRS technologies, especially VR devices, are widely incorporated into education, teaching, and training in various application domains. VRs are useful for many aspects of “soft” skills acquisition, such as cognitive skills, spatial and visual information, visual scanning or observational skills, and affective skills related to controlling emotional responses to stressful or difficult situations (130). Moreover, VR, MR, or HoloLens, *via* visualizing 3D anatomies, could provide medical training programs, such as surgical technique, catheter placement, or cardiopulmonary resuscitation (131–133). The VRS technologies can also create disaster scenarios based on real-world events, and inexperienced students could learn the details of disaster response without being in dangerous situations. Although many authors treated VR or HoloLens as promising learning tools for higher education, only some design-oriented studies based on systematic target theories are constructed (23). Although the procedure- or practice-oriented content, i.e., realistic surrounding teaching environments, made HMDs more exciting and appealing, the actual usability and performance of students using HMDs remained primarily experimental. Only 2.6% of a scoping review study revealed that conceptual frameworks or theories were designed in medical students' VR education (131). Finally, when considering the use of HMDs, we should consider what goals are attainable with them, which technologies are predisposed to incorporate them, and what steps are required to implement them. Although most participants enjoyed and engaged in these novel techniques, realistic HMDs could not replace traditional methods for providing curriculum content in a short time (42). However, with the improvement of new HMD technologies, the wide-range web capacity, cybersickness-prevented design, and cordless HMDs, the new learning modalities will gradually reduce the technological limitations and make the new learning modalities more popular in the future.

We presented the visualized graph drawn by VOSviewer to stress the connection between various keywords, which mirrored the popular study fields and the progress changing with time. However, we have to point out that VOSviewer could not integrate the search results from different databases. The visualized graph was drawn from the literature searched on either PubMed or WES websites. Other limitations are the option methods of VOSviewer to draw the graph, which can affect the visualized result. For example, choosing the keywords defined by the author or MeSH keywords can result in a different view of the graph.

## Conclusion

In this scoping review, by reviewing the state-of-the-art technologies in the last two decades, we found the potential implementation of MECs, such as UAVs combined with IoT, cloud sensor networks, and machine learning was well-suited for integration into emergency services for disaster management. In the future, DM education will be more likely to use blended AI-modulated remote learning models, such as well-developed HMD optical devices based on AI-enhanced real-world scenarios (Figure 4). Moreover, for students to learn well using remote technologies, there should be real-time individual feedback, interactive curriculum review, and AI-assessed algorithms.

## Data availability statement

The original contributions presented in the study are included in the article/Supplementary material, further inquiries can be directed to the corresponding authors.

## Author contributions

C-LK contributed to the conception and design of the study. L-CC organized the database. C-LK and C-CC reviewed all related articles and decided to include extended articles in the study. M-CW and J-ST revised the references. P-CH analyzed and drafted the figures and table. C-LS supervised the study. All authors contributed to the article and approved the submitted version.

## References

[1] Song X Zhang HR Akerkar R Huang HW Guo S Zhong L . Big data and emergency management: concepts, methodologies, and applications. IEEE Trans Big Data. (2022) 8:397–419. 10.1109/tbdata.2020.2972871

[2] Arabi YM Azoulay E Al-Dorzi HM Phua J Salluh J Binnie A . How the COVID-19 pandemic will change the future of critical care. Intensive Care Med. (2021) 47:282–91. 10.1007/s00134-021-06352-y 33616696 PMC7898492

[3] Asadzadeh A Pakkhoo S Saeidabad MM Khezri H Ferdousi R. Information technology in emergency management of COVID-19 outbreak. Inform Med Unlocked. (2020) 21:100475. 10.1016/j.imu.2020.100475 33204821 PMC7661942

[4] Davies S Pettersson T Oberg M. Organized violence 1989-2021 and drone warfare. J Peace Res. (2022) 59:593–610. 10.1177/00223433221108428

[5] Yuan YP Tan GWH Ooi KB Lim WL. Can COVID-19 pandemic influence experience response in mobile learning? Telemat Inform. (2021) 64:101676. 10.1016/j.tele.2021.101676 34887616 PMC8481156

[6] Camilleri MA Camilleri AC. Remote learning *via* video conferencing technologies: implications for research and practice. Technol Soc. (2022) 68:101881. 10.1016/j.techsoc.2022.101881 35034998 PMC8743284

[7] Adedoyin OB Soykan E. Covid-19 pandemic and online learning: the challenges and opportunities. Interact Learn Environ. (2020) 41:1–13. 10.1080/10494820.2020.1813180

[8] Williams KM Corwith A. Beyond bricks and mortar: the efficacy of online learning and community-building at College Park Academy during the COVID-19 pandemic. Educ Inform Technol. (2021) 26:5055–76. 10.1007/s10639-021-10516-0 33814960 PMC8011371

[9] Ray JM Wong AH Yang TJ Buck S Joseph M Bonz JW . Virtual telesimulation for medical students during the COVID-19 pandemic. Acad Med. (2021) 96:1431–5. 10.1097/ACM.0000000000004129 33883398 PMC8475640

[10] Ndzinisa N Dlamini R. Responsiveness vs. accessibility: pandemic-driven shift to remote teaching and online learning. High Educ Res Dev. (2022) 41:1−16. 10.1080/07294360.2021.2019199

[11] UCLouvain C USAID. 2021 Disasters in Numbers-Extreme Events Defining our Lives CRED. Brussels (2022).

[12] CRED Crunch 66 - Disasters Year in Review 2021 [Internet]. USAID. (2022). Available online at: https://www.cred.be/publications (accessed April 2022).

[13] CRED Crunch 64 - Extreme weather events in Europe [Internet]. USAID. (2021). Available online at: https://cred.be/sites/default/files/CredCrunch64.pdf (accessed Spetember 2021).

[14] Liu YL Dai HN Wang QB Imran M Guizani N. Wireless powering Internet of Things with UAVs: challenges and opportunities. IEEE Netw. (2022) 36:146–52. 10.1109/MNET.013.2000385

[15] Kelly JT Campbell KL Gong EY Scuffham P. The Internet of Things: impact and implications for health care delivery. J Med Internet Res. (2020) 22:e20135. 10.2196/20135 33170132 PMC7685921

[16] Abbas N Zhang Y Taherkordi A Skeie T. Mobile edge computing: a survey. IEEE Internet Things J. (2018) 5:450–65. 10.1109/JIOT.2017.2750180

[17] Hu YC Patel M Sabella D Sprecher N Young V. Mobile Edge Computing: A Key Technology Towards 5G. France: ETSI (2015). Report No.: 1–16.

[18] Abrar M. Ajmal Ua, Almohaimeed ZM, Gui X, Akram R, Masroor R. Energy efficient UAV-enabled mobile edge computing for IoT devices: a review. IEEE Access. (2021) 9:127779–98. 10.1109/ACCESS.2021.3112104

[19] Munawar HS Mojtahedi M Hammad AWA Kouzani A Mahmud MAP. Disruptive technologies as a solution for disaster risk management: a review. Sci Total Environ. (2022) 806:151351. 10.1016/j.scitotenv.2021.151351 34740667

[20] Xu J Ota K Dong M. Big data on the fly: UAV-mounted mobile edge computing for disaster management. IEEE Trans Netw Sci Eng. (2020) 7:2620–30. 10.1109/TNSE.2020.3016569

[21] Bruzzone A Longo F Massei M Nicoletti L Agresta M Di Matteo R . Disasters and emergency management in chemical and industrial plants: drones simulation for education and training. In: MESAS 2016: Proceedings of the Third International Workshop on Modelling and Simulation for Autonomous Systems - Volume 9991. Rome: Springer-Verlag (2016). p. 301–8. 10.1007/978-3-319-47605-6_25

[22] Di Giovanni D Fumian F Chierici A Bianchelli M Martellucci L Carminati G . Design of miniaturized sensors for a mission-oriented UAV application: a new pathway for early warning. Int J of Saf Secur Eng. (2021) 11:435–44. 10.18280/ijsse.110417

[23] Radianti J Majchrzak TA Fromm J Wohlgenannt I. A systematic review of immersive virtual reality applications for higher education: design elements, lessons learned, and research agenda. Comput Educ. (2020) 147:103778. 10.1016/j.compedu.2019.103778

[24] Khanal S Medasetti US Mashal M Savage B Khadka R. Virtual and augmented reality in the disaster management technology: a literature review of the past 11 years. Front Virtu Real. (2022) 3:843195. 10.3389/frvir.2022.843195

[25] Feng Z González VA Mutch C Amor R Cabrera-Guerrero G. Instructional mechanisms in immersive virtual reality serious games: earthquake emergency training for children. J Comput Assist Learn. (2020) 37:542–56. 10.1111/jcal.12507

[26] Andreatta PB Maslowski E Petty S Shim W Marsh M Hall T . Virtual reality triage training provides a viable solution for disaster-preparedness. Acad Emerg Med. (2010) 17:870–6. 10.1111/j.1553-2712.2010.00728.x 20670325

[27] Feng Z González VA Trotter M Spearpoint M Thomas J Ellis D . How people make decisions during earthquakes and post-earthquake evacuation: using verbal protocol analysis in immersive virtual reality. Saf Sci. (2020) 129:104837. 10.1016/j.ssci.2020.104837

[28] Xu X Mangina E Campbell AG. HMD-based virtual and augmented reality in medical education: a systematic review. Front Virtual Real. (2021) 2:692103. 10.3389/frvir.2021.69210334255668

[29] Barteit S Lanfermann L Bärnighausen T Neuhann F Beiersmann C. Augmented, mixed, and virtual reality-based head-mounted devices for medical education: systematic review. JMIR Serious Games. (2021) 9:e29080. 10.2196/29080 34255668 PMC8299342

[30] Voicescu GT Valente M Della Corte F Becerril M Ragazzoni L Caviglia M. Medical students' education in disaster medicine: a systematic literature review of existing curricula. Int J Disaster Risk Reduct. (2022) 77:103090. 10.1016/j.ijdrr.2022.10309036676096

[31] Ewing LA Cooper HB. Technology-enabled remote learning during COVID-19: perspectives of Australian teachers, students and parents. Technol Pedagogy Educ. (2021) 30:41–57. 10.1080/1475939X.2020.1868562

[32] Pandey VK De S. Energy-efficient remote mobile device management in infrastructure-less environment for emergency assessment. Int J Commun Syst. (2021) 34:e4930. 10.1002/dac.4930

[33] Ciano JD Acerra J Tang A. Development of a remote learning educational model for international Emergency Medicine trainees in the era of COVID-19. Int J Emerg Med. (2022) 15:1–6. 10.1186/s12245-021-00405-1 34991459 PMC8733921

[34] Potutan G Arakida M. Evolving disaster response practices during COVID-19 pandemic. Int J Environ Res Public Health. (2021) 18:3137. 10.3390/ijerph18063137 33803695 PMC8002954

[35] Feng Y Cui S. A review of emergency response in disasters: present and future perspectives. Nat Hazards. (2020) 105:1109–38. 10.1007/s11069-020-04297-x

[36] Bajow N Djalali A Ingrassia PL Ragazzoni L Ageely H Bani I . Evaluation of a new community-based curriculum in disaster medicine for undergraduates. BMC Med Educ. (2016) 16:225. 10.1186/s12909-016-0746-6 27562428 PMC5000399

[37] Ingrassia PL Ragazzoni L Tengattini M Carenzo L Della Corte F. Nationwide program of education for undergraduates in the field of disaster medicine: development of a core curriculum centered on blended learning and simulation tools. Prehosp Disaster Med. (2014) 29:508–15. 10.1017/S1049023X14000831 25155942

[38] Liu Q Peng WJ Zhang F Hu R Li YG Yan WR. The effectiveness of blended learning in health professions: systematic review and meta-analysis. J Med Internet Res. (2016) 18:e2. 10.2196/jmir.4807 26729058 PMC4717286

[39] Singh J Steele K Singh L. Combining the best of online and face-to-face learning: hybrid and blended learning approach for COVID-19, post vaccine, & post-pandemic world. J Educ Technol Syst. (2021) 50:140–71. 10.1177/00472395211047865

[40] Shakarami A Ghobaei-Arani M Shahidinejad A. A survey on the computation offloading approaches in mobile edge computing: a machine learning-based perspective. Comput Netw. (2020) 182:107496. 10.1016/j.comnet.2020.107496

[41] Stromberga Z Phelps C Smith J Moro C. Teaching with disruptive technology: the use of augmented, virtual, and mixed reality (HoloLens) for disease education. Adv Exp Med Biol. (2021) 1317:147–62. 10.1007/978-3-030-61125-5_8 33945136

[42] Moro C Phelps C Redmond P Stromberga Z. HoloLens and mobile augmented reality in medical and health science education: a randomised controlled trial. Br J Educ Technol. (2020) 52:680–94. 10.1111/bjet.13049

[43] Yazid Y Ez-Zazi I Guerrero-González A El Oualkadi A Arioua M. UAV-enabled mobile edge-computing for IoT based on AI: a comprehensive review. Drones. (2021) 5:148. 10.3390/drones5040148

[44] Blease C Kharko A Bernstein M Bradley C Houston M Walsh I . Machine learning in medical education: a survey of the experiences and opinions of medical students in Ireland. BMJ Health Care Inform. (2022) 29:e100480. 10.1136/bmjhci-2021-100480 35105606 PMC8808371

[45] Mendo IR Marques G. de la Torre Diez I, Lopez-Coronado M, Martin-Rodriguez F. Machine learning in medical emergencies: a systematic review and analysis. J Med Syst. (2021) 45:88. 10.1007/s10916-021-01762-3 34410512 PMC8374032

[46] Yadav SS Jadhav SM. Deep convolutional neural network based medical image classification for disease diagnosis. J Big Data. (2019) 6:113. 10.1186/s40537-019-0276-2

[47] Sufian A Ghosh A Sadiq AS Smarandache F. A survey on deep transfer learning to edge computing for mitigating the COVID-19 Pandemic. J Syst Archit. (2020) 108:101830. 10.1016/j.sysarc.2020.101830

[48] Aledhari M Razzak R Parizi RM Saeed F. Federated learning: a survey on enabling technologies, protocols, and applications. IEEE Access. (2020) 8:140699–725. 10.1109/ACCESS.2020.3013541 32999795 PMC7523633

[49] Tehseen R Farooq MS Abid A. A framework for the prediction of earthquake using federated learning. Peer J Comput Sci. (2021) 7:e540. 10.7717/peerj-cs.540 34141879 PMC8176529

[50] Dusse F Júnior PS Alves AT Novais R Vieira V Mendonça M. Information visualization for emergency management: a systematic mapping study. Expert Syst Appl. (2016) 45:424–37. 10.1016/j.eswa.2015.10.007

[51] Fan J Gao Y Zhao N Dai R Zhang H Feng X . Bibliometric analysis on COVID-19: a comparison of research between English and Chinese studies. Front Public Health. (2020) 8:477. 10.3389/fpubh.2020.00477 32923422 PMC7456831

[52] Du HS Ke X Chu SKW Chan LT. A bibliometric analysis of emergency management using information systems (2000-2016). Online Inf Rev. (2017) 41:454–70. 10.1108/OIR-05-2017-0142

[53] van Eck NJ Waltman L. Software survey: VOSviewer, a computer program for bibliometric mapping. Scientometrics. (2010) 84:523–38. 10.1007/s11192-009-0146-3 20585380 PMC2883932

[54] Sweileh WM. A bibliometric analysis of health-related literature on natural disasters from 1900 to 2017. Health Res Policy Syst. (2019) 17:18. 10.1186/s12961-019-0418-1 30744641 PMC6371570

[55] Hamidah I Sriyono S Nur Hudha M. A bibliometric analysis of Covid 19 research using VOSviewer. Indonesian J Sci Technol. (2020) 5:18. 10.17509/ijost.v5i2.2452236452061

[56] Cobo MJ López-Herrera AG Herrera-Viedma E Herrera F. Science mapping software tools: review, analysis, and cooperative study among tools. J Am Soc Inf Sci Technol. (2011) 62:1382–402. 10.1002/asi.2152535967367

[57] Moher D Liberati A Tetzlaff J Altman DG. Preferred reporting items for systematic reviews and meta-analyses: the PRISMA statement. BMJ. (2009) 339:b2535. 10.1136/bmj.b2535 19622551 PMC2714657

[58] Tricco AC Lillie E Zarin W O'Brien KK Colquhoun H Levac D . PRISMA extension for scoping reviews (PRISMA-ScR): checklist and explanation. Ann Intern Med. (2018) 169:467–73. 10.7326/M18-0850 30178033

[59] Buus L Georgsen M. A learning design methodology for developing short learning programmes in further and continuing education. J Interact Media Educ. (2018) 2018:1–10. 10.5334/jime.469

[60] Means B Toyama Y Murray R Baki M. The effectiveness of online and blended learning: meta-analysis of the empirical literature. Teach Coll Rec. (2013) 115:030303. 10.1177/01614681131150030728150177

[61] Bhattacharya S Singh A Semwal J Marzo RR Sharma N Goyal M . Impact of a training program on disaster preparedness among paramedic students of a tertiary care hospital of North India: a single-group, before-after intervention study. J Educ Health Promot. (2020) 9:5. 10.4103/jehp.jehp_704_19 32154300 PMC7032020

[62] Liu F Guo Y Cai Z Xiao N Zhao Z. Edge-enabled disaster rescue. ACM Trans Intell Syst Technol. (2019) 10:1–21. 10.1145/3331146

[63] Sakurai M Murayama Y. Information technologies and disaster management – benefits and issues. Progress Disaster Sci. (2019) 2:100012. 10.1016/j.pdisas.2019.100012

[64] Li N Sun N Cao C Hou S Gong Y. Review on visualization technology in simulation training system for major natural disasters. Nat Hazards. (2022) 112:1851–82. 10.1007/s11069-022-05277-z 35308193 PMC8923969

[65] Huda SMA Moh S. Survey on computation offloading in UAV-enabled mobile edge computing. J Netw Comput Appl. (2022) 201:103341. 10.1016/j.jnca.2022.103341

[66] Abrahamsen HB. A remotely piloted aircraft system in major incident management: concept and pilot, feasibility study. BMC Emerg Med. (2015) 15:12. 10.1186/s12873-015-0036-3 26054527 PMC4460697

[67] Chuang CC Rau JY Lai MK Shih CL. Combining unmanned aerial vehicles, and internet protocol cameras to reconstruct 3-D disaster scenes during rescue operations. Prehosp Emerg Care. (2019) 23:479–84. 10.1080/10903127.2018.1528323 30260257

[68] Gharahbaghian L Anderson KL Lobo V Huang RW Poffenberger CM Nguyen PD. Point-of-care ultrasound in austere environments: a complete review of its utilization, pitfalls, and technique for common applications in austere settings. Emerg Med Clin North Am. (2017) 35:409–41. 10.1016/j.emc.2016.12.007 28411935

[69] Robin R Murphy ST Alexander K. Disaster robotics. In: Handbook of Robotics, eds B. Siciliano and O. Khatib (Berlin Heidelberg: Springer Nature) (2016), p. 1577–604. 10.1007/978-3-319-32552-1_60

[70] Kryvasheyeu Y Chen H Obradovich N Moro E Van Hentenryck P Fowler J . Rapid assessment of disaster damage using social media activity. Sci Adv. (2016) 2:e1500779. 10.1126/sciadv.1500779 27034978 PMC4803483

[71] Devaraj A Murthy D Dontula A. Machine-learning methods for identifying social media-based requests for urgent help during hurricanes. Intern J Disaster Risk Reduct. (2020) 51:101757. 10.1016/j.ijdrr.2020.101757

[72] Huang CM Chan E. Hyder AA. Web 20_internet social networking: a new tool for disaster management? - lessons from Taiwan. BMC Medical Inform Decis Mak. (2010) 10:5. 10.1186/1472-6947-10-57 20925944 PMC2958996

[73] Daud S Yusof M Heo CC Khoo LS Singh MKC Mahmood MS . Applications of drone in disaster management: a scoping review. Sci Justice. (2022) 62:30–42. 10.1016/j.scijus.2021.11.002 35033326

[74] Pi Y Nath ND Behzadan AH. Convolutional neural networks for object detection in aerial imagery for disaster response and recovery. Adv Eng Inform. (2020) 43:101009. 10.1016/j.aei.2019.101009

[75] Jain T Sibley A Stryhn H Hubloue I. Comparison of unmanned aerial vehicle technology-assisted triage versus standard practice in triaging casualties by paramedic students in a mass-casualty incident scenario. Prehosp Disaster Med. (2018) 33:375–80. 10.1017/S1049023X18000559 30001765

[76] Fernandez-Pacheco AN Rodriguez LJ Price MF Perez ABG Alonso NP Rios MP. Drones at the service for training on mass casualty incident: a simulation study. Medicine. (2017) 96:e7159. 10.1097/MD.0000000000007159 28658106 PMC5500028

[77] James CA Wheelock KM Woolliscroft JO. Machine learning: the next paradigm shift in medical education. Acad Med. (2021) 96:954–57. 10.1097/ACM.0000000000003943 33496428

[78] Kolachalama VB Garg PS. Machine learning and medical education. NPJ Digit Med. (2018) 1:54. 10.1038/s41746-018-0061-1 31304333 PMC6550167

[79] Hartawan DR Purboyo TW Setianingsih C. Disaster victims detection system using convolutional neural network method. In: 2019 IEEE International Conference on Industry 40 Artificial Intelligence and Commuication Technology (IAICT): IEEE Xplore (2019), p. 105–11. 10.1109/ICIAICT.2019.878478234770654

[80] Alzubaidi L Zhang J Humaidi AJ Al-Dujaili A Duan Y Al-Shamma O . Review of deep learning: concepts, CNN architectures, challenges, applications, future directions. J Big Data. (2021) 8:53. 10.1186/s40537-021-00444-8 33816053 PMC8010506

[81] Valdez DB Godmalin RAG. A deep learning approach of recognizing natural disasters on images using convolutional neural network and transfer learning. In: Proceedings of the International Conference on Artificial Intelligence and its Applications Jaipur (2021), p. 1–7. 10.1145/3487923.3487927

[82] Bhattacharya S Reddy Maddikunta PK Pham QV Gadekallu TR Krishnan SS Chowdhary CL . Deep learning and medical image processing for coronavirus (COVID-19) pandemic: a survey. Sustain Cities Soc. (2021) 65:102589. 10.1016/j.scs.2020.102589 33169099 PMC7642729

[83] Yamashita R Nishio M Do RKG Togashi K. Convolutional neural networks: an overview and application in radiology. Insights Imaging. (2018) 9:611–29. 10.1007/s13244-018-0639-9 29934920 PMC6108980

[84] Sarki R Ahmed K Wang H Zhang Y Wang K. Automated detection of COVID-19 through convolutional neural network using chest X-ray images. PLoS ONE. (2022) 17:e0262052. 10.1371/journal.pone.0262052 35061767 PMC8782355

[85] Daniel Shu Wei Ting Lawrence Carin Dzau V Wong TY. Digital technology and COVID-19. Nat Med. (2020) 26:458. 10.1038/s41591-020-0824-5 32284618 PMC7100489

[86] Carin L. On artificial intelligence and deep learning within medical education. Acad Med. (2020) 95:S10–1. 10.1097/ACM.0000000000003630 32769462

[87] Saiboon IM Zahari F Isa HM Sabardin DM Robertson CE. E-learning in teaching emergency disaster response among undergraduate medical students in Malaysia. Front Public Health. (2021) 9:628178. 10.3389/fpubh.2021.628178 33996711 PMC8116625

[88] Sit C Srinivasan R Amlani A Muthuswamy K Azam A Monzon L . Attitudes and perceptions of UK medical students towards artificial intelligence and radiology: a multicentre survey. Insights Imaging. (2020) 11:14. 10.1186/s13244-019-0830-7 32025951 PMC7002761

[89] Wood EA Ange BL Miller DD. Are we ready to integrate artificial intelligence literacy into medical school curriculum: students and faculty survey. J Med Educ Curric Dev. (2021) 8:23821205211024078. 10.1177/23821205211024078 34250242 PMC8239949

[90] Ejaz H McGrath H Wong BL Guise A Vercauteren T Shapey J. Artificial intelligence and medical education: a global mixed-methods study of medical students' perspectives. Digit Health. (2022) 8:1–11. 10.1177/20552076221089099 35521511 PMC9067043

[91] Ishii E Ebner DK Kimura S Agha-Mir-Salim L Uchimido R Celi LA. The advent of medical artificial intelligence: lessons from the Japanese approach. J Intensive Care. (2020) 8:35. 10.1186/s40560-020-00452-5 32467762 PMC7236126

[92] Linardos V Drakaki M Tzionas P Karnavas YL. Machine learning in disaster management: recent developments in methods and applications. Mach Learn Knowl Extra. (2022) 4:446–73. 10.3390/make4020020

[93] Harnessing Earth System Science Technology and Services to Reduce Disaster Risk-WMO Contributions [Internet]. Geneva: WMO (2022).

[94] Nunavath V Goodwin M editors. The Use of AI in Disaster Management- A Systematic Literature Review. (2019) Paris, France: IEEE. 10.1109/ICT-DM47966.2019.903293536873583

[95] Ahmed L Ahmad K Said N Qolomany B Qadir J Al-Fuqaha A. Active learning based federated learning for waste and natural disaster image classification. IEEE Access. (2020) 8:208518–31. 10.1109/ACCESS.2020.3038676

[96] Nguyen DC Ding M Pathirana PN Seneviratne A Li J Poor HV. Federated learning for Internet of Things: a comprehensive survey. IEEE Commun Surv Tutor. (2021) 23:1622–58. 10.1109/COMST.2021.3075439

[97] Zhang Z He N Li D Gao H Gao T Zhou C. Federated transfer learning for disaster classification in social computing networks. J Safety Sci Resilience. (2022) 3:15–23. 10.1016/j.jnlssr.2021.10.007

[98] Xia Q Ye W Tao Z Wu J Li Q. A survey of federated learning for edge computing: research problems and solutions. High Confidence Comput. (2021) 1:100008. 10.1016/j.hcc.2021.10000835062410

[99] Jung YH. Virtual reality simulation for disaster preparedness training in hospitals: integrated review. J Med Internet Res. (2022) 24:e30600. 10.2196/30600 35089144 PMC8838598

[100] Regal G Schrom-Feiertag H Migliorini M Guarneri M Di Giovanni D D'Angelo A . editors. Challenges in virtusl reality training for CRBN events. In: Extended Reality XR Salento 2022 Lecture Notes in Computer Science, Part II; 2022 July 6–8; Lecce, Italy: Springer (2022). 10.1007/978-3-031-15553-6_6

[101] Pottle J. Virtual reality and the transformation of medical education. Future Healthc J. (2019) 181–5. 10.7861/fhj.2019-0036 31660522 PMC6798020

[102] Weiner DL Rosman SL. Just-in-time training for disaster response in the austere environment. Clin Ped Emerg Med. (2019) 20:95–110. 10.1016/j.cpem.2019.07.001

[103] Ragazzoni L Barco A Echeverri L Conti A Linty M Caviglia M . Just-in-time training in a tertiary referral hospital during the COVID-19 Pandemic in Italy. Acad Med. (2021) 96:336–9. 10.1097/ACM.0000000000003575 32639262 PMC7363358

[104] Wahyudin D Hasegawa S editors. The role of serious games in disaster and safety education: an integrative review. In: 25th International Conference on Computers in Education. New Zealand (2017).27885969

[105] IJgosse W van Goor H Rosman C Luursema JM. Construct validity of a serious game for laparoscopic skills training: validation study. JMIR Serious Games. (2020) 8:e17222. 10.2196/17222 32379051 PMC7243133

[106] Olgers TJ. bij de Weg AA, ter Maaten JC. Serious games for improving technical skills in medicine: scoping review. JMIR Serious Games. (2021) 9:e24093. 10.2196/24093 33492234 PMC7870348

[107] Solinska-Nowak A Magnuszewski P Curl M French A Keating A Mochizuki J . An overview of serious games for disaster risk management – prospects and limitations for informing actions to arrest increasing risk. Int J Disaster Risk Reduct. (2018) 31:1013–29. 10.1016/j.ijdrr.2018.09.001

[108] Peek LA. Children and disasters: understanding vulnerability, developing capacities, and promoting resilience- an introduction. Child Youth Environ. (2008) 18:1–29. Available online at: https://www.jstor.org/stable/10.7721/chilyoutenvi.18.1.0001

[109] Mutch C Carol M Dr Jay Marlowe D. “Sailing through a river of emotions”: capturing children's earthquake stories. Disaster Prev Manag. (2013) 22:445–55. 10.1108/DPM-10-2013-0174

[110] Omlor AJ Schwarzel LS Bewarder M Casper M Damm E Danziger G . Comparison of immersive and non-immersive virtual reality videos as substitute for in-hospital teaching during coronavirus lockdown: a survey with graduate medical students in Germany. Med Educ Online. (2022) 27:2101417. 10.1080/10872981.2022.2101417 35850619 PMC9302008

[111] Bhatti I Jones K Richardson L Foreman D Lund J Tierney G. E-learning vs lecture: which is the best approach to surgical teaching? Colorectal Dis. (2011) 13:459–62. 10.1111/j.1463-1318.2009.02173.x 20041922

[112] Huber T Paschold M Hansen C Wunderling T Lang H Kneist W. New dimensions in surgical training: immersive virtual reality laparoscopic simulation exhilarates surgical staff. Surg Endosc. (2017) 31:4472–7. 10.1007/s00464-017-5500-6 28378077

[113] Renganayagalu Sk Mallam SC Nazir S. Effectiveness of VR head mounted displays in professional training: a systematic review. Technol Knowl Learn. (2021) 26:999–1041. 10.1007/s10758-020-09489-9

[114] Ashcroft J Byrne MHV Brennan PA Davies RJ. Preparing medical students for a pandemic: a systematic review of student disaster training programmes. Postgrad Med J. (2021) 97:368–79. 10.1136/postgradmedj-2020-137906 32518075 PMC7316122

[115] Shaw R. Thirty years of science, technology, and academia in disaster risk reduction and emerging responsibilities. Int J Disaster Risk Sci. (2020) 11:414–25. 10.1007/s13753-020-00264-z

[116] Martin F Ndoye A Wilkins P. Using learning analytics to enhance student learning in online courses based on quality matters standards. J Educ Technol Syst. (2016) 45:165–87. 10.1177/0047239516656369

[117] Álvarez-García C Cámara-Anguita S López-Hens JM Granero-Moya N López-Franco MD María-Comino-Sanz I . Development of the aerial remote triage system using drones in mass casualty scenarios: a survey of international experts. PLoS ONE. (2021) 16:e0242947. 10.1371/journal.pone.0242947 33974634 PMC8112676

[118] Ray PP Mukherjee M Shu L. Internet of things for disaster management: state-of-the-art and prospects. IEEE Access. (2017) 5:18818–35. 10.1109/ACCESS.2017.2752174

[119] Javed F Afzal MK Sharif M Kim BS. Internet of things (IoT) operating systems support, networking technologies, applications, and challenges: a comparative review. IEEE Commun Surv Tutor. (2018) 20:2062–100. 10.1109/COMST.2018.281768535812486

[120] Miraz MH Ali M Excell PS Picking R editors. A review on Internet of Things (IoT), Internet of Everything (IoE) and Internet of Nano Things (IoNT). In: Internet Technol Appl (ITA). Wrexham, UK: IEEE (2015). 10.1109/ITechA.2015.731739836905010

[121] Balasubramaniam S Kangasharju J. Realizing the internet of nano things challenges solutions and applications. Computer. (2013) 46:62–8. 10.1109/MC.2012.389

[122] Kortuem G Bandara AK Smith N Richards M Petre M. Educating the Internet-of-Things generation. Computer. (2013) 46:53–61. 10.1109/MC.2012.390

[123] Arinta RR Andi WRE. Natural disaster application on big data and machine learning: a review. In: 2019 4th International Conference on Information Technology, Information Systems and Electrical Engineering (ICITISEE). (2019), p. 249–54. 10.1109/ICITISEE48480.2019.9003984

[124] Sarker MNI Peng Y Yiran C Shouse RC. Disaster resilience through big data: way to environmental sustainability. Int J Disaster Risk Reduct. (2020) 51:101769. 10.1016/j.ijdrr.2020.101769

[125] Joseph JK Dev KA Pradeepkkumar AP Mohan M. Big data analytics and social media in disaster management. In:Samui P Kim D Ghosh C, editors. Integrating Disaster Science and Management: Global Case Studies in Mitigation and Recovery. Netherlands: Candice Janco. (2018), p. 287–94.

[126] Moreira F Ferreira MJ Cardoso A editors. Higher education disruption through IoT and Big Data: a conceptual approach. In: Learning and Collaboration Techologies: Novel Learning Ecosystems; 2017 08 June. Vancouver, BC: Springer, 389–405. 10.1007/978-3-319-58509-3_31

[127] Klemke R Eradze M Antonaci A. The flipped MOOC: using gamification and learning analytics in MOOC design- a conceptual approach. Educ Sci. (2018) 8:25–38. 10.3390/educsci8010025

[128] Al-Balas M Al-Balas HI Jaber HM Obeidat K Al-Balas H Aborajooh EA . Distance learning in clinical medical education amid COVID-19 pandemic in Jordan: current situation, challenges, and perspectives. BMC Med Educ. (2020) 20:341. 10.1186/s12909-020-02257-4 33008392 PMC7530879

[129] Chan KS Zary N. Applications and challenges of implementing artificial intelligence in medical education: integrative review. JMIR Med Educ. (2019) 5:e13930. 10.2196/13930 31199295 PMC6598417

[130] Jensen L Konradsen F. A review of the use of virtual reality head-mounted displays in education and training. Educ Inform Technol. (2017) 23:1515–29. 10.1007/s10639-017-9676-0

[131] Jiang H Vimalesvaran S Wang JK Lim KB Mogali SR Car LT. Virtual reality in medical students' education: scoping review. JMIR Med Educ. (2022) 8:e34860. 10.2196/34860 35107421 PMC8851326

[132] Schoeb DS Schwarz J Hein S Schlager D Pohlmann PF Frankenschmidt A . Mixed reality for teaching catheter placement to medical students: a randomized single-blinded, prospective trial. BMC Med Educ. (2020) 20:510. 10.1186/s12909-020-02450-5 33327963 PMC7745503

[133] Wong M Chue S Jong M Benny HWK Zary N. Clinical instructors' perceptions of virtual reality in health professionals' cardiopulmonary resuscitation education. SAGE Open Med. (2018) 6:1–8. 10.1177/2050312118799602 30245815 PMC6144504

