# Supplementary figures and images for: The development of new remote technologies in disaster medicine education: A scoping review
Source: Front Public Health. 2023 Mar 24;11:1029558. doi: 10.3389/fpubh.2023.1029558 (PMC10080133; doi:10.3389/fpubh.2023.1029558)

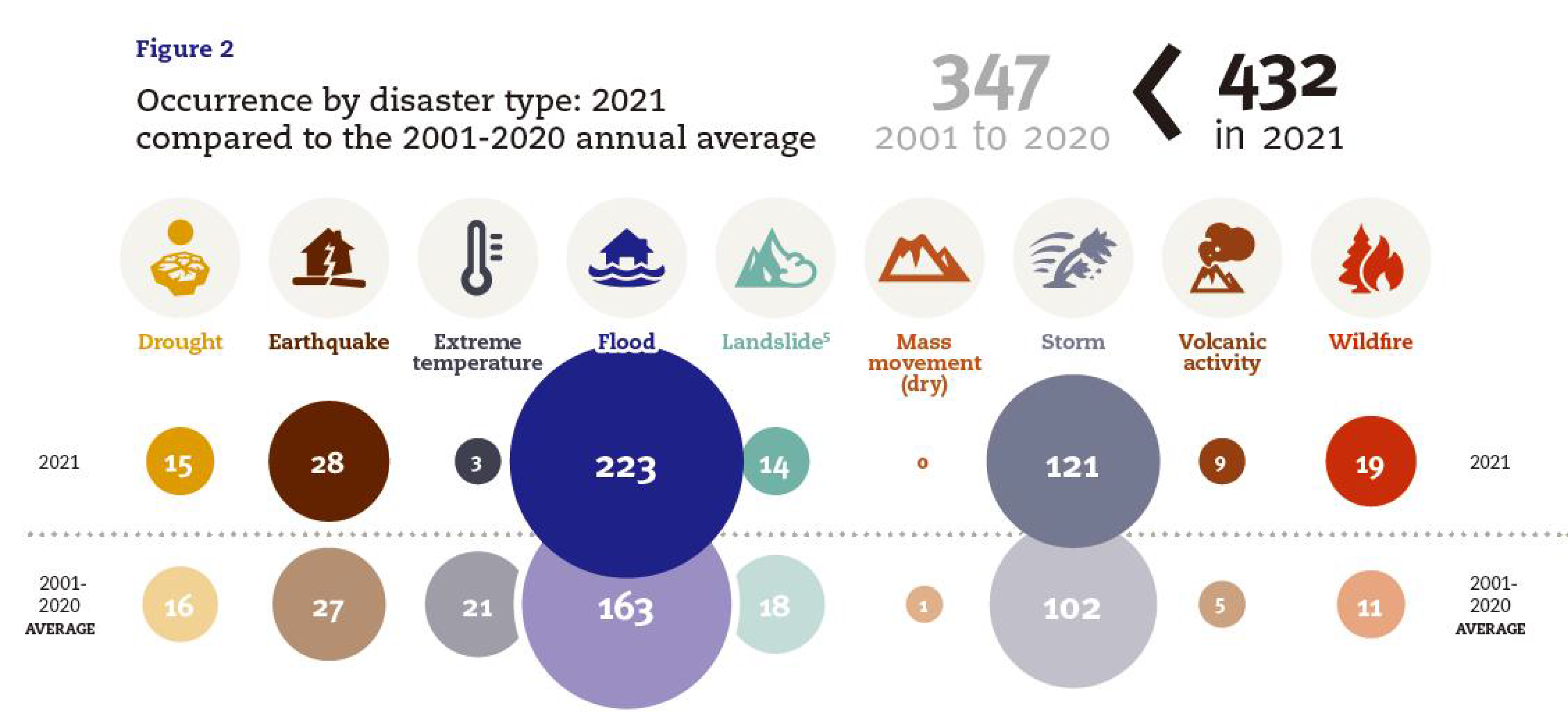

Supplement: Supplementary file 2 [file Image_1.TIF]
